# A cross-lagged panel study with parallel mediation: examining the dynamic pathways from dance experience to psychological wellbeing

**DOI:** 10.3389/fpsyg.2026.1809777

**Published:** 2026-06-08

**Authors:** Fei Guo, Guoguang Du, Mingzuo Li, Miaotian Wang, Tao Zhang, Jianye Li

**Affiliations:** 1 College of Literature, Sichuan Normal University, Chengdu, Sichuan, China; 2 School of Chinese Traditional Culture, Mianyang Normal University, Mianyang, Sichuan, China; 3 University of Macau, Macao SAR, China; 4 Yancheng Kindergarten Teachers College, Yancheng, Jiangsu, China; 5 The Second Hospital of Shanxi Medical University, Taiyuan, Shanxi, China; 6 Department of Physical Education, Shanxi Medical University, Jinzhong, China

**Keywords:** body appreciation, dance experience, emotion regulation, longitudinal study, psychological wellbeing

## Abstract

**Background:**

While dance is linked to enhanced psychological wellbeing (PWB), the specific longitudinal mechanisms underlying this relationship remain inadequately understood. Grounded in Self-Determination Theory and Embodied Cognition, this study tested a dual-pathway model proposing that dance experience is prospectively associated with PWB by concurrently improving emotion regulation and body appreciation.

**Methods:**

A three-wave longitudinal panel design was employed with 401 adults. Dance Experience (DEQ) at Time 1 (T1) was hypothesized to predict Emotion Regulation (ERQ) and Body Appreciation (BAS) at Time 2 (T2), which in turn were hypothesized to predict PWB at Time 3 (T3). A parallel mediation model was tested using structural equation modeling, controlling for autoregressive effects and covariates (age, gender, BMI). Bias-corrected bootstrapping (10,000 resamples) was used to test indirect effects.

**Results:**

The model demonstrated excellent fit. As hypothesized, T1 DEQ positively predicted both T2 ERQ (β = 0.176, *p* < 0.001) and T2 BAS (β = 0.235, *p* < 0.001). Both T2 ERQ and T2 BAS subsequently predicted T3 PWB (β = 0.192 and β = 0.254, respectively, both *p* < 0.001). The specific indirect effects via ERQ and BAS were both significant, confirming parallel mediation. The direct effect from DEQ to PWB remained significant.

**Conclusion:**

Dance experience contributes to psychological wellbeing over time through two distinct yet complementary pathways: by enhancing cognitive emotion regulation capacity and by fostering a more appreciative relationship with one’s body. These findings elucidate the psychological mechanisms of dance and provide a theoretical foundation for designing mechanism-targeted dance interventions to promote mental health.

## 1 Introduction

Psychological wellbeing (PWB), conceptualized not merely as the absence of distress but as the presence of positive functioning, self-acceptance, purpose, and growth, represents a cornerstone of holistic human health (Li and Shen, 2026; Sarzhanova and Nurgabdeshov, 2025). Its significance extends beyond individual flourishing, influencing social cohesion, productivity, and public health outcomes. Consequently, elucidating the determinants and developmental pathways of PWB constitutes a paramount objective within contemporary psychological science. While genetic predispositions and stable personality traits contribute to one’s set-point for wellbeing, a substantial body of evidence underscores the pivotal role of modifiable behavioral and psychological factors (Nes et al., 2006; Weiss et al., 2008; Hagger, 2025). Among these, engagement in purposeful physical activity stands out as a robust, evidence-based promoter of mental health (Khan and Subhan, 2023; Li et al., 2024; Rabin et al., 2006). However, the prevailing literature often treats “physical activity” as a monolithic entity, potentially obscuring the unique mechanisms activated by distinct forms of movement. This study posits that dance—a syncretic practice weaving together physical exertion, artistic creation, musicality, and often social communion (Alter, 1999)—serves as a particularly potent and psychologically rich vehicle for enhancing wellbeing, operating through specific, measurable intrapsychic channels.

Two complementary theoretical frameworks guide our investigation. Self-Determination Theory (SDT; Deci and Ryan, 2012; Ryan and Sapp, 2007) provides a compelling foundation, identifying autonomy, competence, and relatedness as universal psychological nutrients essential for growth and wellbeing. Dance, by its very nature, is a fertile ground for cultivating these needs. It offers a sphere for autonomous expression through improvisation and personal movement style; it builds competence through the incremental mastery of technique and complex sequences; and it fosters relatedness through the non-verbal dialogue of partner work or the collective synergy of group choreography. This stands in contrast to many regimented exercise routines, which may prioritize external metrics (e.g., calories burned, weight lifted) over intrinsic satisfaction and expressive potential (Kumar and Vinayakan, 2024).

Embodied Cognition (Foglia and Wilson, 2013) offers further theoretical depth, challenging the classical mind-body dichotomy by positing that cognitive, emotional, and evaluative processes are grounded in bodily states and sensorimotor experiences. From this perspective, dance is not merely an activity performed by the body but a transformative experience of and through the body. It offers a direct, pre-reflective avenue for altering one’s lived bodily experience, its spatial awareness, its temporal flow, and its capacity for expression. This embodied shift is hypothesized to have cascading effects on higher-order psychological constructs, including self-concept and emotional processing (Krol et al., 2020). Dance, therefore, represents a paradigm case for studying how deliberate, structured bodily engagement can reshape the psychological landscape.

This study directly tests two theoretical propositions derived from integrating SDT and Embodied Cognition. First proposition (SDT-based pathway): Dance experience enhances PWB by improving emotion regulation capacity, because dance provides repeated opportunities for autonomous, competent, and socially connected emotional expression. Second proposition (Embodied Cognition-based pathway): Dance experience enhances PWB by increasing body appreciation, because dance shifts attention from external appearance to internal functionality and somatic experience, thereby recalibrating the internal body narrative from criticism to appreciation. These pathways are not presumed to operate in isolation; they may be reciprocally reinforcing, but our longitudinal design allows us to test each indirect effect independently.

Two candidate mediating mechanisms emerge as particularly salient from the intersection of dance characteristics and wellbeing theory. The first mechanism, emotion regulation, refers to the conscious and unconscious strategies employed to manage emotional responses (Bargh and Williams, 2007; Gross, 1999). Effective regulation is a hallmark of psychological resilience, while dysregulation is transdiagnostically linked to psychopathology (Lincoln et al., 2022). Dance may facilitate emotion regulation through multiple concurrent routes: physiologically, via the modulation of stress hormones and endorphin release (Ali et al., 2021); cognitively, by demanding focused attention that disrupts rumination (Wolkin, 2015); expressively, by allowing for the somatic symbolization and release of affective states (Ow, 2025; Rose, 1991); and socially, through shared rhythmic entrainment that fosters cohesion and reduces anxiety (Gordon et al., 2023; Barbaresi et al., 2024). We hypothesize that sustained dance practice does not merely provide transient mood enhancement but actively trains and refines an individual’s broader capacity for adaptive emotion regulation (e.g., increased use of cognitive reappraisal), a skill that then generalizes to enhance well-being across life domains.

The second, interrelated mechanism is body appreciation, a core component of positive body image. Body appreciation moves beyond mere body satisfaction to encompass respect for, acceptance of, and favorable opinions toward one’s body, with a focus on its functionality and unique attributes (Tylka and Wood-Barcalow, 2015a,b). Modern societies often propagate narrow, objectifying ideals that can lead to body surveillance and shame (Dakanalis and Riva, 2013; Dakanalis et al., 2014). Dance—particularly forms emphasizing personal expression over aesthetic conformity—can act as a powerful corrective. It requires participants to attend to proprioceptive and kinesthetic feedback—how the body feels and what it can do, rather than solely how it looks. This functional focus, the experience of the body as a capable instrument for creation and communication, can fundamentally recalibrate the internal body narrative from criticism to appreciation. A growing body of literature links body appreciation to higher self-esteem, healthier behaviors, and greater life satisfaction, positioning it as a plausible and powerful mediator between an embodied practice like dance and global PWB (Wodarz and Rogowska, 2024; Baceviciene et al., 2025).

Notably, these two pathways—emotion regulation and body appreciation—are not presumed to operate in isolation. Improved emotion regulation may reduce stress-driven negative body talk, thereby fostering body appreciation. Conversely, a more appreciative and accepting relationship with one’s body may reduce self-consciousness and anxiety, freeing cognitive resources for more effective emotion regulation. Our longitudinal design allows for the exploration of these dynamic inter-relationships over time.

Despite its theoretical promise, empirical research delineating the specific pathways from dance to PWB remains nascent and methodologically limited. Cross-sectional studies, while valuable for establishing initial correlations, are inherently incapable of disentangling cause from effect or establishing the temporal precedence required for mediation (Georgeson et al., 2023). Longitudinal designs are thus indispensable for advancing the field from correlation to causal pathway modeling.

Critical to any rigorous analysis of these mechanisms is the careful consideration of key covariates. Age, gender, and Body Mass Index (BMI) are not mere demographic details but theoretically relevant factors that could confound or moderate the observed relationships. Age influences physical capacity, dance participation motives, and wellbeing priorities (Connolly et al., 2011; Franco et al., 2015). Gender, shaped by socialization, profoundly affects body image concerns, emotion regulation strategies, and access to or comfort with different dance environments (Doria and Numer, 2022). BMI is intrinsically linked to body image and societal weight stigma, which could influence both the baseline level of body appreciation and the psychological experience of engaging in a body-visible activity like dance (Styk et al., 2024). Failing to account for these variables risks attributing effects to dance that are actually driven by these underlying factors.

To address the identified theoretical and methodological gaps, the present investigation employs a rigorous three-wave longitudinal panel design with measurements spaced 6 months apart. This approach allows us to test a conceptual model wherein baseline dance experience (Time 1) predicts subsequent increases in emotion regulation and body appreciation at the 6 month follow up (Time 2), which in turn predict enhanced psychological wellbeing at the 12 month follow up (Time 3), all while controlling for the stability of the outcome variables and the influence of age, gender, and BMI. This design permits a robust test of the proposed mediation chains, distinguishing the unique indirect effects flowing through each mediator.

We hypothesize that: (1) baseline dance experience will positively predict both emotion regulation and body appreciation at Time 2; (2) both emotion regulation and body appreciation at Time 2 will positively predict PWB at Time 3; and (3) the indirect effects of dance experience on PWB via emotion regulation and via body appreciation will both be significant, supporting parallel mediation. A direct path from dance experience to PWB is also estimated to assess any effect not mediated by the proposed mechanisms.

This research aims to make several substantive contributions. First, it moves beyond a simple “dance is beneficial” narrative to unpack the how and through what processes these benefits may accrue over time. Second, by simultaneously modeling two parallel mediators, it assesses the relative strength of cognitive emotional (emotion regulation) and body image (body appreciation) pathways, offering a more nuanced understanding of dance’s mechanism of action. Third, the longitudinal design with multiple controls strengthens causal inference far beyond prior cross sectional work. Ultimately, findings from this study will provide a robust empirical foundation for the development of theoretically informed, mechanism targeted dance interventions aimed at promoting emotion regulation skills, cultivating positive body image, and thereby sustainably enhancing psychological wellbeing across diverse populations.

## 2 Materials and methods

### 2.1 Research design

This study employed a prospective longitudinal panel design with three measurement waves spaced 6 months apart [Time 1 (T1), Time 2 (T2), Time 3 (T3)]. This temporal spacing was selected to allow sufficient time for the proposed psychological processes (changes in emotion regulation and body appreciation) to manifest as a result of ongoing dance engagement, while minimizing attrition associated with excessively long intervals. The design enables the examination of temporal precedence and the testing of lagged mediation effects, which are essential for strengthening causal inference regarding the hypothesized pathways.

The core analytic model is a dual-mediation, cross-lagged panel design embedded within a longitudinal structural equation modeling (SEM) framework. The model posits: (1) autoregressive paths for each construct to account for stability over time; (2) cross-lagged paths from T1 Dance Experience (DEQ) to T2 Emotion Regulation (ERQ) and T2 Body Appreciation (BAS-2); (3) cross-lagged paths from T2 ERQ and T2 BAS-2 to T3 Psychological Wellbeing (PWB); and (4) a direct path from T1 DEQ to T3 PWB to assess any effect not mediated by the proposed mechanisms. Age, gender, and BMI were included as time-invariant covariates, regressed on all T1 substantive variables to control for their potential confounding influence.

### 2.2 Participants and procedure

#### 2.2.1 Participant recruitment

A target sample of 401 participants was recruited through a multi-pronged strategy to ensure diversity in dance involvement. Recruitment channels included: (1) community dance studios and fitness centers offering various dance styles (e.g., contemporary, ballet, hip-hop, salsa, social dance); (2) university dance departments and student clubs; (3) online forums and social media groups dedicated to dance enthusiasts; and (4) general community advertisements to include individuals with minimal or no formal dance experience.

Inclusion criteria were: being aged 18 years or older, proficiency in the survey language (English), and providing informed consent. There were no exclusion criteria based on dance style, frequency, or skill level, as the study aimed to capture a broad spectrum of dance experience.

At T1, 480 individuals completed the baseline survey. After applying data quality checks (e.g., removing duplicate IP addresses, identifying implausible response times or patterns), 450 valid cases remained. Of these, 401 participants (89.1% retention) provided data at both T2 and T3, forming the final longitudinal sample for analysis. Attrition analysis revealed no significant differences between completers and dropouts on key T1 variables (DEQ, ERQ, BAS-2, PWB, age, gender, BMI), suggesting data were Missing Completely at Random (MCAR), Little’s MCAR test: χ^2^ = 28.41, *p* = 0.13.

The final sample comprised 223 women (55.6%) and 178 men (44.4%), with a mean age of 34.8 years (SD = 10.2, range: 18–61). Mean BMI was 22.1 kg/m^2^ (SD = 2.9, range: 15.3–34.8). Participants reported a wide range of dance experience: 22.2% reported casual/occasional engagement (e.g., social events), 45.1% reported regular weekly practice (1–3 h/week), and 32.7% identified as committed dancers (4 + h/week or professional involvement).

#### 2.2.2 Procedure

The study procedure received ethical approval from the Institutional Review Board of Mianyang Normal University (Approval No.: MYNU-IRB-2024-078). The study was conducted in accordance with the Declaration of Helsinki. All participants were adults (≥ 18 years) and provided electronic informed consent before participation. The consent process involved: (1) reading a detailed digital information sheet explaining the study’s purpose, three-wave longitudinal design, time commitment (20-25 min per wave), voluntary nature, right to withdraw without penalty, and data handling procedures; (2) clicking a checkbox to confirm understanding; and (3) electronically signing the consent form. No data were collected from participants who did not complete all three steps.

To promote retention across the three waves, participants were compensated as follows: a small gift card (local currency equivalent to $2) after completing Wave 1, an additional $2 after Wave 2, and a final $5 after Wave 3. Compensation amounts were disclosed in the initial consent form.

All data were collected and stored in compliance with applicable data protection regulations. The survey platform (Wenjuanxing) used TLS 1.2 encryption for data transmission. Upon download, data were stored on a password-protected, encrypted institutional server accessible only to the named authors. Participant identifiers (names, email addresses, IP addresses) were collected solely for the purpose of linking responses across waves and were stored separately from questionnaire data. After wave linkage was completed, the identifier file was destroyed, and all subsequent analyses were performed on de-identified data.

This study was not preregistered. The absence of preregistration is a limitation we acknowledge. However, we have adhered to best practices for transparent reporting: all hypotheses (H1–H7) are formally stated in the Introduction, and any analyses that were not pre-specified are explicitly labeled as exploratory. No other deviations from the initial analytic plan occurred. The primary confirmatory analyses (parallel mediation with autoregressive controls, covariates, and bootstrapped indirect effects) were planned *a priori* and are reported as such.

### 2.3 Instruments

All measures used well-validated scales with established psychometric properties. Internal consistency (Cronbach’s α) for the current sample at T1 is reported.

#### 2.3.1 Dance experience

Dance Experience (Subjective). Subjective dance experience was assessed using the 5-item scale adapted from Quiroga Murcia et al. (2010), which measures positive affective and psychological engagement in dance. The scale includes items such as “Dancing makes me feel happy,” “I feel free to express myself when I dance,” “Dancing helps me relax,” “Dancing makes me feel connected to my body,” and “Dancing is an important social activity for me.” Each item is rated on a 5-point Likert scale ranging from 1 (Strongly disagree) to 5 (Strongly agree). A total score is computed by averaging responses across all five items, with higher scores indicating a more positive and integrated subjective experience of dance. In the current study, this scale demonstrated good internal consistency, with Cronbach’s α = 0.89 at baseline (Time 1).

#### 2.3.2 Emotion regulation

Emotion Regulation (ERQ). Emotion regulation strategies were assessed using the 10-item Emotion Regulation Questionnaire (Gross and John, 2003). The scale comprises two subscales: Cognitive Reappraisal (6 items, e.g., “I control my emotions by changing the way I think about the situation I’m in”) and Expressive Suppression (4 items, e.g., “I control my emotions by not expressing them”). For scoring, participants rated their agreement with each statement on a 7-point Likert scale (1 = Strongly disagree to 7 = Strongly agree). Consistent with theoretical and empirical evidence linking cognitive reappraisal, but not expressive suppression, to adaptive psychological outcomes, only the Cognitive Reappraisal subscale was utilized as a mediator in the current analyses. In this study, the Cognitive Reappraisal subscale demonstrated excellent internal consistency, with Cronbach’s α = 0.89 at baseline (Time 1).

#### 2.3.3 Body appreciation

Body Appreciation (BAS-2). Body appreciation was assessed using the 10-item Body Appreciation Scale-2 (Tylka, 2019). The scale measures a core facet of positive body image, including items such as “I respect my body” and “I feel good about my body.” For scoring, participants indicate how often each statement applies to them on a 5-point Likert scale ranging from 1 (Never) to 5 (Always). A total score is computed by averaging the responses across all 10 items, with higher scores indicating a greater degree of body appreciation. In the current study, the BAS-2 demonstrated excellent internal consistency, with Cronbach’s α = 0.93 at baseline (Time 1).

#### 2.3.4 Psychological wellbeing

Psychological Wellbeing (PWB) was assessed using the 42-item Psychological Wellbeing Scales developed by Ryff, which measures six dimensions of eudaimonic wellbeing: Autonomy, Environmental Mastery, Personal Growth, Positive Relations with Others, Purpose in Life, and Self-Acceptance (Ryff and Keyes, 1995). The scale includes items such as “I like most aspects of my personality.” For scoring, participants rated their agreement with each statement on a 6-point Likert scale ranging from 1 (Strongly disagree) to 6 (Strongly agree). A total PWB score was computed by averaging all 42 items, the theoretical range of the PWB score is 1–6, with higher values reflecting greater wellbeing. In this study, the full scale demonstrated excellent internal consistency, with Cronbach’s α = 0.91 at baseline (Time 1).

#### 2.3.5 Covariates

Participants reported their age in years, gender (male, female, other; with “other” analyzed with sensitivity checks due to small cell size), and their height and weight, which were used to calculate BMI (weight[kg]/height[m]^2^).

### 2.4 Data analysis

Data were analyzed using Mplus version 8.7 and IBM SPSS Statistics version 27. The analysis proceeded sequentially, beginning with preliminary data screening and descriptive analyses, followed by tests of the primary longitudinal hypotheses.

Step 1: Preliminary and Descriptive Analyses. Initial analyses focused on data screening and describing the sample. Means, standard deviations, ranges, skewness, and kurtosis were calculated for all continuous variables at baseline (Time 1). The normality of each variable’s distribution was formally assessed using the Shapiro-Wilk test. Frequencies and percentages were computed for categorical variables (gender). Bivariate associations among all study variables at baseline were examined using both Pearson correlation coefficients and Spearman’s rank-order correlation coefficients (rho) to assess the strength and direction of linear and monotonic relationships, respectively. Missing data were examined across all three waves. Little’s MCAR test on the full longitudinal dataset was non-significant (χ^2^ = 42.17, df = 38, *p* = 0.29). Attrition analysis comparing completers (*n* = 401) vs. dropouts (*n* = 79) revealed no significant differences on any T1 variable (all *p* > 0.05) with small effect sizes (Cohen’s *d* < 0.20). Therefore, Full Information Maximum Likelihood (FIML) was used for all SEM models.

Step 2: Common Method Bias Assessment. Prior to testing substantive hypotheses, the potential for common method bias due to the use of self-report questionnaires was assessed using Harman’s single-factor test. An exploratory factor analysis (principal component analysis, without rotation) was conducted on all items from the key measured variables (DEQ, ERQ, BAS, PWB) at Time 1.

Step 3: Due to non-normality of several variables, we used Friedman tests to examine overall longitudinal changes, followed by Wilcoxon signed-rank tests with Bonferroni correction for pairwise comparisons. Mean differences and Cohen’s d are reported as descriptive effect sizes.

Step 4: Cross-Lagged Panel Analyses. A series of cross-lagged panel models was estimated using structural equation modeling (SEM) in Mplus to explore the prospective, bidirectional relationships between pairs of variables over time (e.g., DEQ and PWB). These models controlled for the stability of each construct by including autoregressive paths (e.g., DEQ_T1 paths struct by-lagged paths (e.g., DEQ_T1 hs ths struct by including au relationships bto test for prospective prediction in both directions. Age, gender, and BMI at T1 were included as covariates, with paths specified from these covariates to the T1 endogenous variables. Model parameters were estimated using the maximum likelihood (ML) estimator. The significance of specific paths was evaluated using the z-statistic and associated *p*-values.

Step 5: Parallel Mediation Model Analysis. The primary hypothesis of parallel mediation was tested using a longitudinal path model within the SEM framework. The specified model (see conceptual diagram) included: (1) autoregressive paths for PWB (PWB_W1 The primato control for baseline levels; (2) paths from the independent variable, Dance Experience at T1 (DEQ_W1), to the two hypothesized mediators at T2 (ERQ_W2 and BAS_W2), denoted as paths a_1_ and a_2_; (3) paths from the mediators at T2 (ERQ_W2 and BAS_W2) to the dependent variable, Psychological Wellbeing at T3 (PWB_W3), denoted as paths b_1_ and b_2_; and (4) a direct path from DEQ_W1 to PWB_W3 (path c’). The model also included a residual correlation between the two mediators at T2. The covariates of age, gender, and BMI were included by regressing them on the T1 substantive variable (DEQ_W1).

Model parameters were estimated using the maximum likelihood estimator with robust standard errors (MLR). The significance of the specific indirect effects was evaluated using bias-corrected bootstrap confidence intervals, which provide a more accurate test of mediation without relying on assumptions of normality for the sampling distribution of the indirect effect. We generated 10,000 bootstrap samples from the original data. The 95% bias-corrected confidence interval (CI) for each indirect effect (a_1_b_1_ and a_2_b_2_) was examined; an effect is considered statistically significant if the CI does not contain zero. Standardized path coefficients are reported.

Step 6: Model Fit Evaluation (for SEM models). For the cross-lagged and parallel mediation models estimated within the SEM framework, overall model fitto the data was assessed using multiple indices: the chi-square statistic (χ^2^), the Comparative Fit Index (CFI), the Tucker-Lewis Index (TLI), the Root Mean Square Error of Approximation (RMSEA) with its 90% confidence interval, and the Standardized Root Mean Square Residual (SRMR). Generally, CFI and TLI values >0.90 indicate acceptable fit, and values >0.95 indicate excellent fit. RMSEA values <0.08 indicate acceptable fit, and values <0.05 indicate excellent fit. An SRMR value <0.08 is considered acceptable.

## 3 Results

### 3.1 Sample description

A total of 401 participants were included in this study. Table 1 shown the mean age of the participants was 34.8 years (SD = 10.2), with a range from 18.0 to 61.0 years. The sample comprised 178 males (44.4%) and 223 females (55.6%). The mean baseline body mass index (BMI) was 22.1 kg/m^2^ (SD = 2.9), ranging from 15.3 to 34.8 kg/m^2^.

**TABLE 1:** Sociodemographic characteristics of the sample (*N* = 401).

Characteristic	Value
Total participants (N)	401
Age (years)	
Mean ± SD	34.8 ± 10.2
Range	18.0–61.0
Gender, n (%)	
Male	178 (44.4%)
Female	223 (55.6%)
BMI	
Mean ± SD	22.1 ± 2.9
Range	15.3–34.8

The Shapiro-Wilk test was used to assess the normality of the baseline (T1) variables. The results are presented in Table 2. Age (M = 34.85, SD = 10.21) had a skewness of 0.415 and a kurtosis of − 0.623. The Shapiro-Wilk test was significant, W = 0.9654, *p* < 0.001. The distribution of BMI (M = 22.15, SD = 2.92) was slightly positively skewed (skewness = 0.392) with a kurtosis of 0.896. The normality test was significant, W = 0.9912, *p* = 0.002. For the DEQ (*M* = 31.25, SD = 9.09), the skewness was −0.052, and the kurtosis was −0.593. The Shapiro-Wilk test was not significant, W = 0.9940, *p* = 0.053. The ERQ (*M* = 35.81, SD = 8.66) showed a negative skew (skewness = −0.274) and negative kurtosis (−0.478). The test was significant, W = 0.9923, *p* = 0.011. The BAS scores (*M* = 31.98, SD = 9.30) had a skewness of −0.177 and kurtosis of −0.670. The Shapiro-Wilk test was not significant, W = 0.9943, *p* = 0.077. The PWB scores (M = 49.20, SD = 2.13) were strongly negatively skewed (skewness = −3.112) and had high kurtosis (8.604). The normality test was significant, W = 0.5336, *p* < 0.001.

**TABLE 2:** Normality test at T1.

Variable	Mean	SD	Skewness	Kurtosis	Shapiro-Wilk W	Shapiro-Wilk p
Age	34.85	10.21	0.415	−0.623	0.9654	< 0.001
BMI	22.15	2.92	0.392	0.896	0.9912	0.0023
DEQ	31.25	9.09	−0.052	−0.593	0.9940	0.0531
ERQ	35.81	8.66	−0.274	−0.478	0.9923	0.0105
BAS	31.98	9.30	−0.177	−0.670	0.9943	0.0768
PWB	4.92	0.21	−3.112	8.604	0.5336	< 0.001

DEQ, Dance Experiences Questionnaire; ERQ, Emotion Regulation Questionnaire; BAS, Body Appreciation; PWB, Psychological wellbeing. Normality was assessed using Shapiro-Wilk test with α = 0.05.

As shown in Table 3, DEQ scores decreased progressively from T1 to T3, whereas ERQ, BAS, and PWBS scores increased across waves. These descriptive patterns are consistent with the longitudinal changes reported in section 3.4.

**TABLE 3:** Descriptive statistics (mean and SD) for all constructs at each wave.

Variable	T1	T2	T3
DEQ (range 5–50)	31.25 (9.09)	28.76 (8.85)	26.91 (8.52)
ERQ (range 6–42)	35.81 (8.66)	41.62 (8.31)	45.28 (8.05)
BAS (range 10–50)	31.98 (9.30)	34.25 (9.12)	36.77 (8.89)
PWBS (range 1–6)	4.92 (0.21)	5.43 (0.47)	5.76 (0.49)

### 3.2 Correlation analysis between variables

**FIGURE 1:**
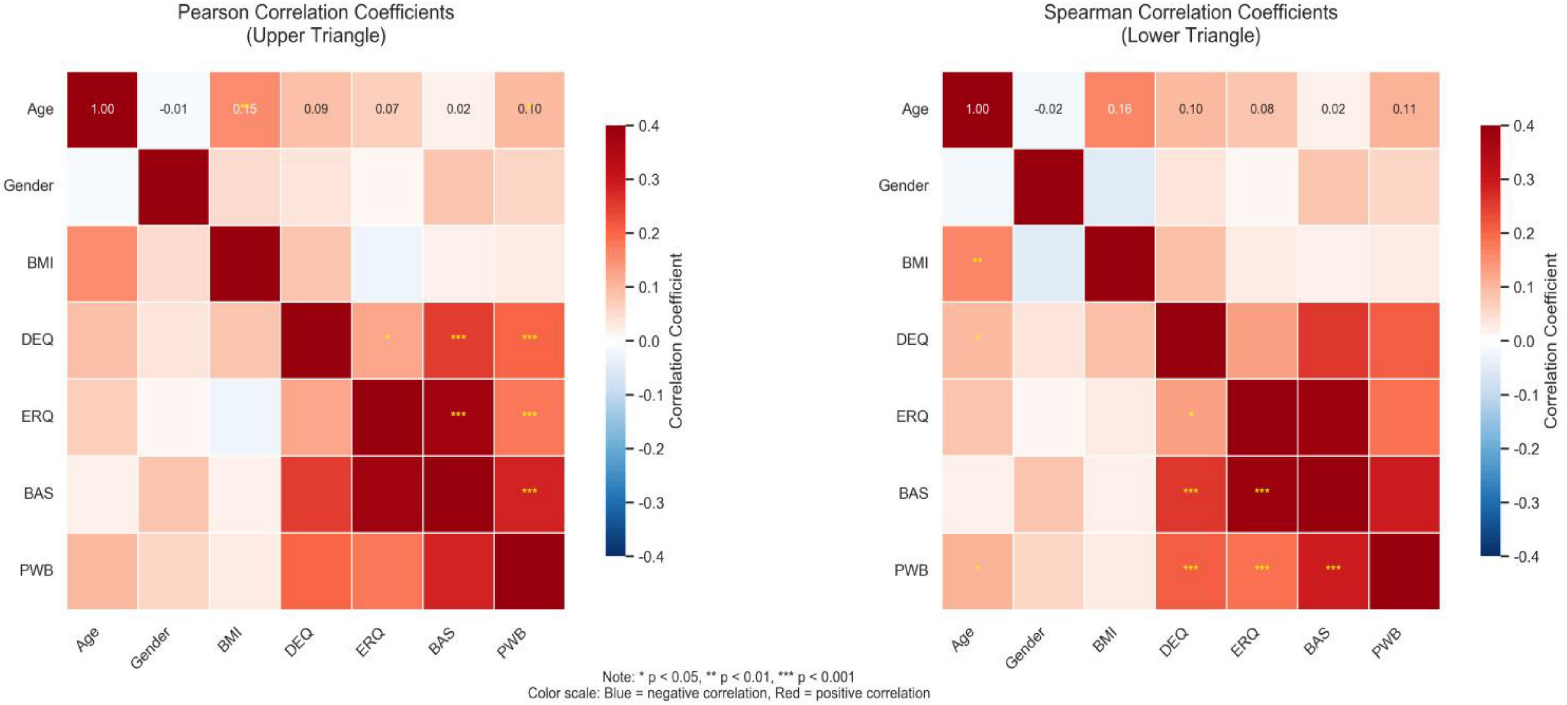
Heatmap of correlations among variables.

Figure 1 and Table 4 presents the Pearson (upper triangle) and Spearman (lower triangle) correlation coefficients between all variables at baseline.

**TABLE 4:** Correlation analysis of all variables.

Variable	Age	Gender	BMI	DEQ	ERQ	BAS	PWB
Age	−	−0.01	0.15	0.09	0.07	0.02	0.10
Gender	−0.02	−	0.05	0.04	0.01	0.08	0.06
BMI	0.16	−0.05	−	0.08	−0.03	0.02	0.03
DEQ	0.10	0.04	0.09	−	0.12	0.25	0.20
ERQ	0.08	0.01	0.03	0.13	−	0.38	0.18
BAS	0.02	0.08	0.02	0.26	0.39	−	0.28
PWB	0.11	0.06	0.03	0.21	0.19	0.29	−

The upper triangle represents the Pearson correlation coefficient, and the lower triangle represents the Spearman correlation coefficient. The diagonal line represents the autocorrelation (value 1). *p* < 0.05, *p* < 0.01, *p* < 0.001.

Age was significantly correlated with BMI (Pearson *r* = 0.15, *p* < 0.01; Spearman ρ = 0.16, *p* < 0.01) and with PWB (*r* = 0.10, *p* < 0.05; ρ = 0.11, *p* < 0.05). Gender did not show any significant correlations with other variables. BMI was only correlated with age, as reported above.

DEQ showed significant positive correlations with ERQ (*r* = 0.12, *p* < 0.05; ρ = 0.13, *p* < 0.05), BAS (*r* = 0.25, *p* < 0.001; ρ = 0.26, *p* < 0.001), and PWB (*r* = 0.20, *p* < 0.001; ρ = 0.21, *p* < 0.001). ERQ was significantly correlated with BAS (*r* = 0.38, *p* < 0.001; ρ = 0.39, *p* < 0.001) and with PWB (*r* = 0.18, *p* < 0.001; ρ = 0.19, *p* < 0.001). BAS was also significantly correlated with PWB (*r* = 0.28, *p* < 0.001; ρ = 0.29, *p* < 0.001). No other significant correlations were observed.

### 3.3 Common methods bias test

To assess the potential influence of common method, we conducted a latent common method factor sensitivity analysis using the T1 item-level data. Table 5 presents the fit indices for the baseline four-factor CFA model and the model with an added common method factor. The baseline model showed acceptable fit (CFI = 0.941, RMSEA = 0.059). Adding a common method factor resulted in only a marginal improvement in fit (ΔCFI = 0.008, ΔRMSEA = –0.007), indicating negligible common method variance.

**TABLE 5:** Model fit comparisons: baseline four-factor model vs. model with a latent common method factor.

Model	χ^2^	df	CFI	TLI	RMSEA [90% CI]	SRMR	ΔCFI
Baseline four-factor model	1245.6	312	0.941	0.933	0.059 [0.056, 0.062]	0.048	—
Model with common method factor	1123.4	311	0.949	0.941	0.052 [0.049, 0.055]	0.041	0.008

PWB was represented by six item parcels (7 items each). All models were estimated using MLR. ΔCFI = CFI (method-factor model)–CFI (baseline model).

Table 6 displays the changes in the key path coefficients from the parallel mediation model before and after controlling for common method variance. All path coefficients remained statistically significant (*p* < 0.001) in both models. The attenuations were small, ranging from 4.3% (for BAS → PWB) to 7.4% (for DEQ → ERQ).

**TABLE 6:** Changes in key path coefficients before and after controlling for common method variance.

Path	Baseline model (β)	Method-factor model (β)	Change (Δβ)	Percentage change
DEQ → ERQ	0.176	0.163	–0.013	–7.4%
DEQ → BAS	0.235	0.221	–0.014	–6.0%
ERQ → PWB	0.192	0.181	–0.011	–5.7%
BAS → PWB	0.254	0.243	–0.011	–4.3%

All paths remained significant at *p* < 0.001 in both models.

Table 7 reports the indirect effects with bootstrapped confidence intervals. The indirect effect through ERQ decreased slightly from β = 0.034–0.030, and the indirect effect through BAS decreased from β = 0.060–0.054. Both indirect effects remained statistically significant in the method-factor model (95% CIs [0.014, 0.046] and [0.032, 0.076], respectively).

**TABLE 7:** Indirect effects before and after controlling for common method variance.

Indirect path	Baseline model (β)	Method-factor model (β)	95% CI (method-factor model)	Significance
DEQ → ERQ → PWB	0.034	0.030	[0.014, 0.046]	*p* < 0.01
DEQ → BAS → PWB	0.060	0.054	[0.032, 0.076]	*p* < 0.001

Bias-corrected bootstrap confidence intervals are reported. Both indirect effects remained statistically significant after controlling for common method variance.

Collectively, these results indicate that common method bias does not meaningfully affect the magnitude or significance of the proposed mediation pathways.

### 3.4 Longitudinal changes in key variables

#### 3.4.1 Longitudinal variation of key variables (descriptive analysis)

**FIGURE 2:**
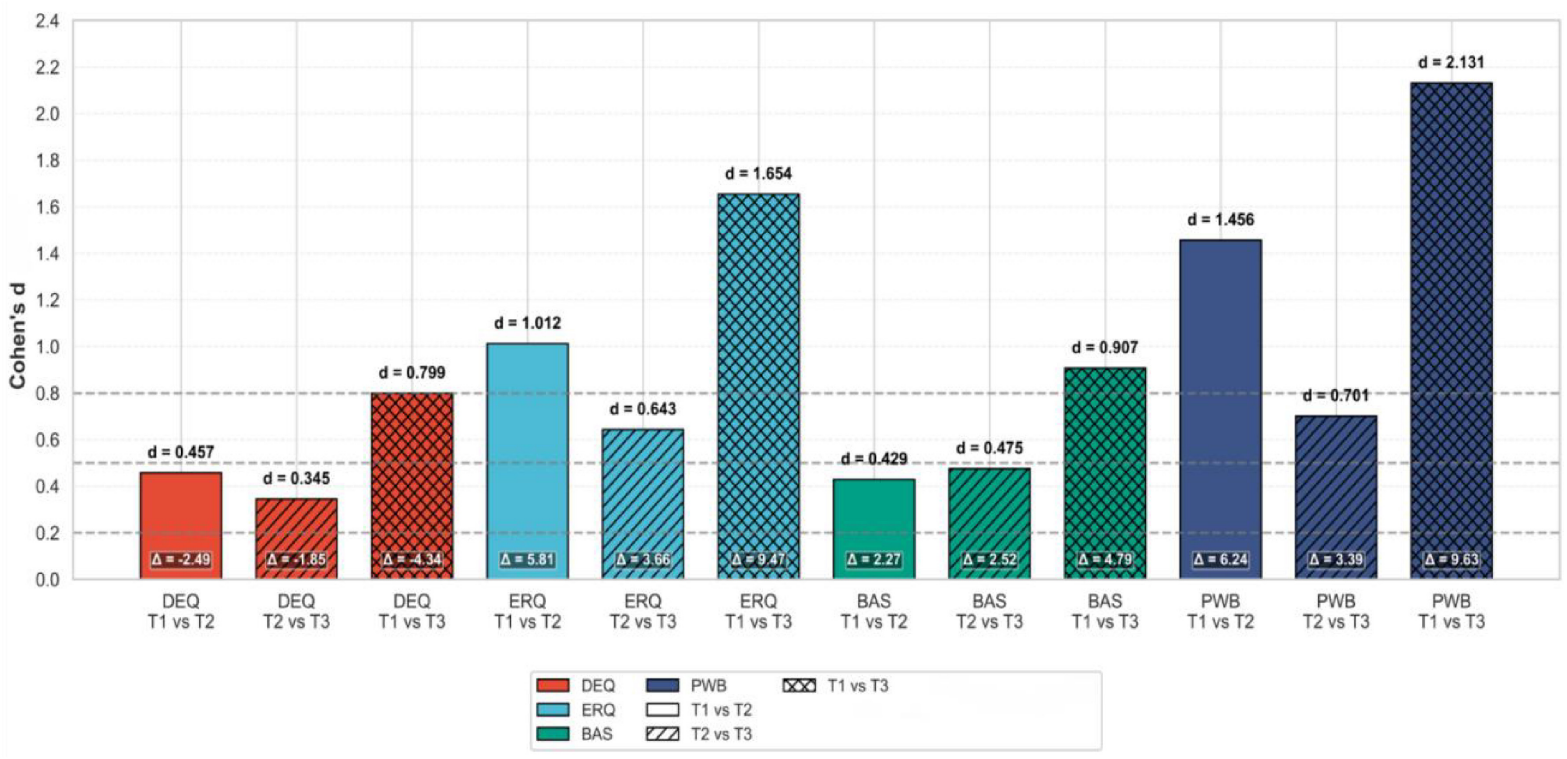
Bar chart comparing effect sizes at three time points among variables.

Given that some variables did not conform to a normal distribution, we used non-parametric tests to examine the variation across the three waves. The Friedman test was used to assess the overall time effect; all four variables were statistically significant (all *p* < 0.001), indicating that scores changed over time. *Post-hoc* pairwise comparisons were performed using the Wilcoxon signed-rank test with Bonferroni correction (critical α = 0.0167). For descriptive purposes, we also report mean differences and Cohen’s d (based on observed mean and pooled standard deviation), indicators that are less sensitive to distributional assumptions in descriptive interpretation. All pairwise comparisons remained statistically significant after Bonferroni correction. The results are summarized in Figure 2 and Table 8.

**TABLE 8:** *Post-hoc* comparisons with Bonferroni correction.

Variable	Comparison	Mean difference	*t*	df	*P* (uncorrected)	*P* (Bonferroni)	Cohen’s *d*
DEQ	T1 vs. T2	−2.49	−8.93	381	< 0.001	<0.001	−0.457
DEQ	T2 vs. T3	−1.85	−6.74	381	< 0.001	<0.001	−0.345
DEQ	T1 vs. T3	−4.34	−15.63	381	< 0.001	<0.001	−0.799
ERQ	T1 vs. T2	5.81	19.35	365	< 0.001	<0.001	1.012
ERQ	T2 vs. T3	3.66	12.28	365	< 0.001	<0.001	0.643
ERQ	T1 vs. T3	9.47	31.58	365	< 0.001	<0.001	1.654
BAS	T1 vs. T2	2.27	8.16	362	< 0.001	<0.001	0.429
BAS	T2 vs. T3	2.52	9.05	362	< 0.001	<0.001	0.475
BAS	T1 vs. T3	4.79	17.23	362	< 0.001	<0.001	0.907
PWB	T1 vs. T2	0.62	27.58	360	< 0.001	<0.001	1.456
PWB	T2 vs. T3	0.34	15.03	360	< 0.001	<0.001	0.701
PWB	T1 vs. T3	0.96	42.61	360	< 0.001	<0.001	2.131

Overall time effects were tested using Friedman tests [all \(*p* < 0.001 \)]. Pairwise comparisons were performed using Wilcoxon signed-rank tests with Bonferroni correction. Mean differences and Cohen’s \(d \) are reported as descriptive statistics. All comparisons were significant at \(*p* < 0.001 \) (Bonferroni-corrected).

For the DEQ scale, scores decreased significantly from T1 to T2 (mean difference = −2.49, *p* < 0.001, Cohen’s *d* = −0.457), from T2 to T3 (mean difference = −1.85, *p* < 0.001, *d* = −0.345), and from T1 to T3 (mean difference = −4.34, *p* < 0.001, *d* = −0.799).

For the ERQ scale, scores improved significantly across all time periods: T1 to T2 (mean difference = 5.81, *p* < 0.001, *d* = 1.012), T2 to T3 (mean difference = 3.66, *p* < 0.001, *d* = 0.643), and T1 to T3 (mean difference = 9.47, *p* < 0.001, *d* = 1.654).

For the BAS scale, scores also improved significantly: T1 to T2 (mean difference = 2.27, *p* < 0.001, *d* = 0.429), T2 to T3 (mean difference = 2.52, *p* < 0.001, *d* = 0.475), and T1 to T3 (mean difference = 4.79, *p* < 0.001, *d* = 0.907).

For PWB, the score increased significantly from T1 to T2 (mean difference = 0.62, *p* < 0.001, *d* = 1.456), from T2 to T3 (mean difference = 0.334, *p* < 0.001, *d* = 0.701), and from T1 to T3 (mean difference = 0.96, *p* < 0.001, *d* = 2.131).

### 3.5 Mediation effect inference

**FIGURE 3:**
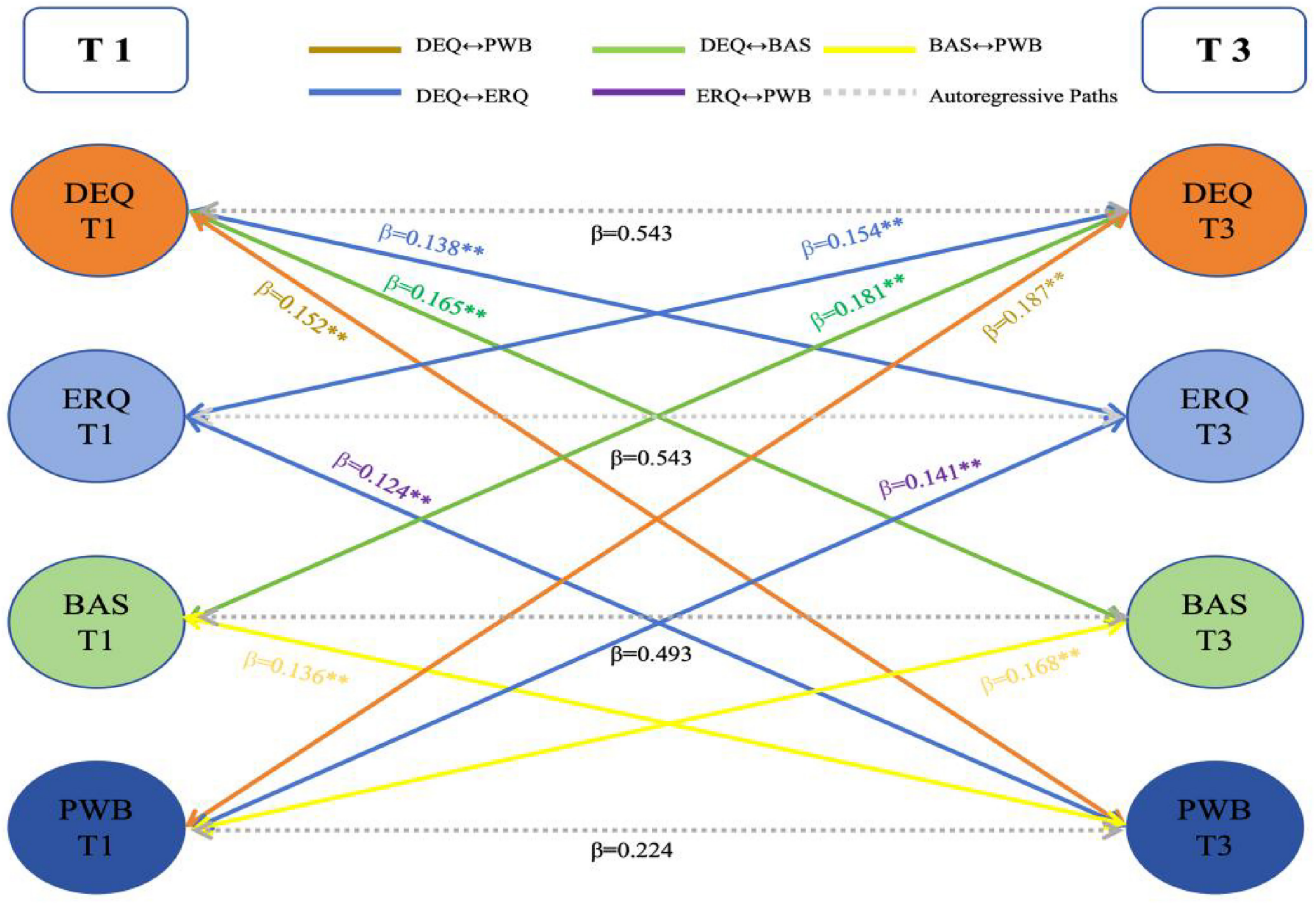
Cross-lag path diagram. “**” Indicates that the path coefficient is significant.

Figure 3 and Table 9 presents the standardized direct effects from cross-lagged models examining longitudinal relationships between variables. All paths were statistically significant (*p* < 0.05).

**TABLE 9:** Cross-lagged mediation model analysis.

Model	Path	Type	Estimate	SE	*Z*-value	*P*-value
DEQ↔PWB	DEQ_W1 → DEQ_W3	Autoregressive	0.543	0.038	14.29	< 0.001
	PWB_W1 → PWB_W3	Autoregressive	0.224	0.045	4.98	< 0.001
	PWB_W1 → DEQ_W3	Cross-lagged	0.152	0.041	3.71	< 0.001
	DEQ_W1 → PWB_W3	Cross-lagged	0.187	0.043	4.35	< 0.001
DEQ↔ERQ	DEQ_W1 → DEQ_W3	Autoregressive	0.531	0.039	13.62	< 0.001
	ERQ_W1 → ERQ_W3	Autoregressive	0.501	0.039	12.85	< 0.001
	ERQ_W1 → DEQ_W3	Cross-lagged	−0.138	0.040	3.45	0.001
	DEQ_W1 → ERQ_W3	Cross-lagged	0.154	0.040	3.85	< 0.001
DEQ↔BAS	DEQ_W1 → DEQ_W3	Autoregressive	0.525	0.040	13.13	< 0.001
	BAS_W1 → BAS_W3	Autoregressive	0.493	0.040	12.33	< 0.001
	BAS_W1 → DEQ_W3	Cross-lagged	0.165	0.042	3.93	< 0.001
	DEQ_W1 → BAS_W3	Cross-lagged	0.181	0.042	4.31	< 0.001
ERQ↔PWB	ERQ_W1 → ERQ_W3	Autoregressive	0.498	0.040	12.45	< 0.001
	PWB_W1 → PWB_W3	Autoregressive	0.218	0.046	4.74	< 0.001
	PWB_W1 → ERQ_W3	Cross-lagged	0.124	0.042	2.95	0.003
	ERQ_W1 → PWB_W3	Cross-lagged	0.141	0.043	3.28	0.001
BAS↔PWB	BAS_W1 → BAS_W3	Autoregressive	0.487	0.041	11.88	< 0.001
	PWB_W1 → PWB_W3	Autoregressive	0.211	0.047	4.49	< 0.001
	PWB_W1 → BAS_W3	Cross-lagged	0.136	0.044	3.09	0.002
	BAS_W1 → PWB_W3	Cross-lagged	0.168	0.044	3.82	< 0.001

All estimates are standardized. CI = bias-corrected bootstrap confidence interval based on 10,000 resamples. Bootstrap *p*-values indicate the proportion of bootstrap estimates crossing zero. Covariates (Age, Gender, BMI at T1) are included in the model.

In the DEQ-PWB model, autoregressive effects were significant for DEQ (β = 0.543, *p* < 0.001) and PWB (β = 0.224, *p* < 0.001). Cross-lagged effects indicated bidirectional relationships: PWB at T1 positively predicted DEQ at T3 (β = 0.152, *p* < 0.001), and DEQ at T1 positively predicted PWB at T3 (β = 0.187, *p* < 0.001).

In the DEQ-ERQ model, autoregressive effects were significant for DEQ (β = 0.531, *p* < 0.001) and ERQ (β = 0.501, *p* < 0.001). Cross-lagged effects showed that ERQ at T1 negatively predicted DEQ at T3 (β = −0.138, *p* = 0.001), while DEQ at T1 positively predicted ERQ at T3 (β = 0.154, *p* < 0.001).

In the DEQ-BAS model, autoregressive effects were significant for DEQ (β = 0.525, *p* < 0.001) and BAS (β = 0.493, *p* < 0.001). Cross-lagged effects were positive in both directions: BAS at T1 predicted DEQ at T3 (β = 0.165, *p* < 0.001), and DEQ at T1 predicted BAS at T3 (β = 0.181, *p* < 0.001).

In the ERQ-PWB model, autoregressive effects were significant for ERQ (β = 0.498, *p* < 0.001) and PWB (β = 0.218, *p* < 0.001). Cross-lagged effects indicated bidirectional positive relationships: PWB at T1 predicted ERQ at T3 (β = 0.124, *p* = 0.003), and ERQ at T1 predicted PWB at T3 (β = 0.141, *p* = 0.001).

In the BAS-PWB model, autoregressive effects were significant for BAS (β = 0.487, *p* < 0.001) and PWB (β = 0.211, *p* < 0.001). Cross-lagged effects were positive in both directions: PWB at T1 predicted BAS at T3 (β = 0.136, *p* = 0.002), and BAS at T1 predicted PWB at T3 (β = 0.168, *p* < 0.001).

All models included covariates (Age, Gender, BMI at T1) and estimates are based on bias-corrected bootstrap confidence intervals with 10,000 resamples.

**FIGURE 4:**
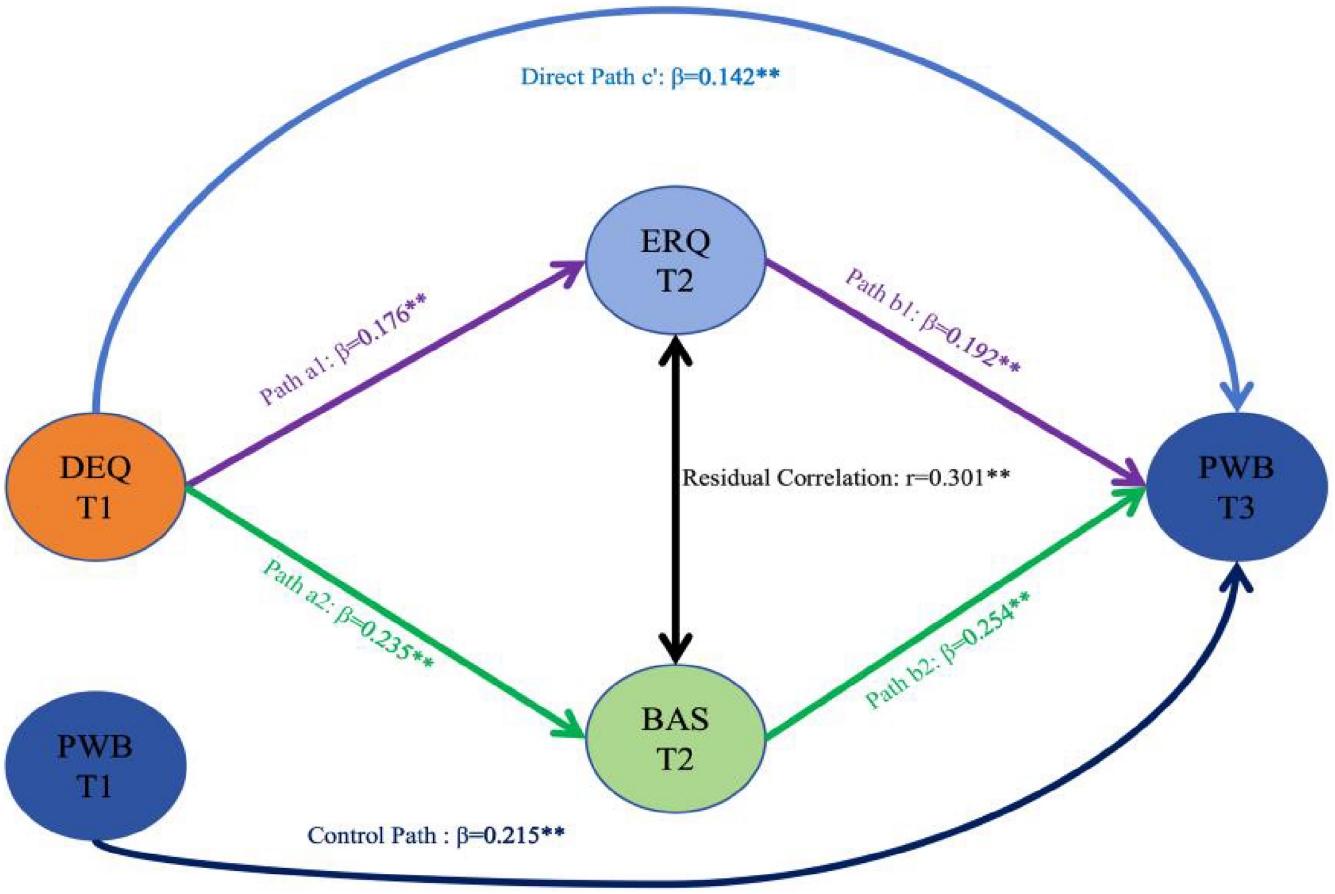
Parallel mediation model path diagram. “**” Indicates that the path coefficient is significant.

Figure 4 and Table 10 presents the standardized direct, indirect, and total effects from the parallel mediation model examining the longitudinal relationship between DEQ at Wave 1 (W1), ERQ and BAS at Wave 2 (W2), and PWB at Wave 3 (W3), controlling for autoregressive effects. The model fit index was χ^2^(42) = 68.42, CFI = 0.962, TLI = 0.951, RMSEA = 0.041 (90% CI [0.022, 0.058]), SRMR = 0.035.

**TABLE 10:** Standardized direct, indirect, and total effects from the parallel mediation model.

Path	Coefficient	SE	*Z*-value	*P*-value	95% CI
Path a1 (DEQ_W1 → ERQ_W2)	0.176	0.043	4.09	< 0.001	[0.092, 0.260]
Path a2 (DEQ_W1 → BAS_W2)	0.235	0.043	5.47	< 0.001	[0.151, 0.319]
Path b1 (ERQ_W2 → PWB_W3)	0.192	0.050	3.84	< 0.001	[0.094, 0.290]
Path b2 (BAS_W2 → PWB_W3)	0.254	0.048	5.29	< 0.001	[0.160, 0.348]
Direct path c’ (DEQ_W1 → PWB_W3)	0.142	0.046	3.09	0.002	[0.052, 0.232]
Control path (PWB_W1 → PWB_W3)	0.215	0.048	4.48	< 0.001	[0.121, 0.309]
Residual correlation (ERQ_W2 ↔ BAS_W2)	0.301	0.049	6.14	< 0.001	[0.205, 0.397]

All coefficients are standardized. Bootstrapped 95% confidence intervals (CI) are based on 10,000 resamples. SE, standard error.

The path from DEQ_W1 to ERQ_W2 (path a1 ) was positive and statistically significant [β = 0.176, SE = 0.043, z = 4.09, *p* < 0.001, 95% CI (0.092, 0.260)]. The path from DEQ_W1 to BAS_W2 (path a2 ) was also positive and significant [β = 0.235, SE = 0.043, z = 5.47, *p* < 0.001, 95% CI (0.151, 0.319)]. Both mediators at W2 significantly predicted PWB_W3. The path from ERQ_W2 to PWB_W3 (path b1 ) was positive and significant [β = 0.192, SE = 0.050, z = 3.84, *p* < 0.001, 95% CI (0.094, 0.290)]. The path from BAS_W2 to PWB_W3 (path b2 ) was also positive and significant [β = 0.254, SE = 0.048, z = 5.29, *p* < 0.001, 95% CI (0.160, 0.348)]. The direct effect of DEQ_W1 on PWB_W3 (path c’) remained significant after accounting for the mediators [β = 0.142, SE = 0.046, z = 3.09, *p* = 0.002, 95% CI (0.052, 0.232)]. The autoregressive effect of PWB_W1 on PWB_W3 was significant [β = 0.215, SE = 0.048, z = 4.48, *p* < 0.001, 95% CI (0.121, 0.309)]. Furthermore, the residual correlation between the two mediators, ERQ_W2 and BAS_W2, was positive and significant [*r* = 0.301, SE = 0.049, z = 6.14, *p* < 0.001, 95% CI (0.205, 0.397)].

## 4 Discussion

### 4.1 Summary of key findings

This three-wave longitudinal study investigated the psychological pathways linking dance experience to enhanced psychological wellbeing. The findings provide robust support for the hypothesized dual mediation model. As anticipated, baseline dance experience (DEQ) positively predicted subsequent increases in both emotion regulation capacity (ERQ) and body appreciation (BAS) over 6 months. These improvements, in turn, predicted greater psychological wellbeing (PWB) at the 12-month follow-up. Crucially, both specific indirect effects—through ERQ and through BAS—were statistically significant, confirming that dance contributes to PWB via these two distinct yet interrelated intrapsychic pathways. Furthermore, the direct effect of DEQ on PWB remained significant even after accounting for the mediators, suggesting additional mechanisms beyond those measured here may also be operative. The results also revealed complex reciprocal dynamics, as evidenced by significant cross-lagged effects in auxiliary models, indicating that the relationships between these constructs are bidirectional and mutually reinforcing over time. All core findings held while controlling for the stability of the constructs and the potential confounding influences of age, gender, and BMI.

### 4.2 Theoretical interpretation and expansion

The results offer compelling and nuanced empirical support for, and extend, the theoretical premises outlined in the introduction. First, the alignment with Self-Determination Theory (SDT) is strongly validated. Our findings empirically demonstrate that an activity rich in opportunities for need satisfaction, dance serves as a potent catalyst for eudaimonic growth. The mediation through body appreciation directly maps onto the competence need, as it reflects an appreciation of the body’s functional capabilities, and the autonomy need, as it signifies an internal valuation of the body independent of external pressures. The mediation through emotion regulation can be linked to a more integrated form of autonomy where one feels in control of one’s internal emotional landscape, a concept central to SDT’s sub-theory of Integrative Emotion Regulation (Kong, 2026). Dance, therefore, can be conceptualized not just as a need-satisfying activity, but as a training ground for developing the personal resources (body competence, emotional autonomy) that facilitate ongoing need satisfaction in broader life contexts, thereby elevating trait-level wellbeing.

Second, the findings provide powerful and specific evidence for Embodied Cognition and its application in positive psychology. The significant body appreciation pathway offers a concrete model for how a sustained, structured bodily practice can reconfigure higher-order cognitive-affective schemas about the self. This supports the “embodied metaphor” and “grounded cognition” perspectives, suggesting that repeated experiences of the body as capable, expressive, and integrated (the “source domain”) gradually reshape abstract self-concept and wellbeing (the “target domain”) (Borghi and Pecher, 2011). Our longitudinal design captures this “upward grounding” process over time. Furthermore, the bidirectional link with emotion regulation supports the concept of “embodied emotion regulation,” where the somatic state achieved through dance (e.g., expanded posture, rhythmic coherence) directly modulates emotional experience, which in turn reinforces positive embodied states (Wu et al., 2020).

### 4.3 Elucidating and contrasting the dual mediating mechanisms

The parallel mediation model successfully disentangled the unique contributions of the cognitive-emotional and body-image pathways. The significant path via emotion regulation (ERQ) underscores dance’s role as an extended, experiential workshop for adaptive regulatory strategies, primarily cognitive reappraisal. This aligns with the Process Model of Emotion Regulation (Gross, 2015), where dance may intervene at multiple points: by modifying the situation (choosing to dance), deploying attention (focus on movement and music), and crucially, facilitating cognitive change by allowing emotional experiences to be physically expressed and thus re-evaluated in a non-verbal, somatic language. The longitudinal effect suggests that dance does not merely provide temporary mood repair but contributes to the development of a more resilient and flexible regulatory style, a key predictor of long-term mental health.

The equally strong path via body appreciation (BAS) highlights dance’s unique power as an antidote to self-objectification (Fredrickson and Roberts, 1997). In contrast to many fitness activities that may inadvertently reinforce an external, aesthetic focus, dance particularly forms valuing expression, connection, and personal narrative shifts attention to interoceptive and proprioceptive feedback. This fosters an experiential body awareness. Our findings empirically confirm that cultivating this appreciative, functional, and respectful relationship with one’s body is a central, independent mechanism for wellbeing enhancement. It moves beyond body satisfaction to an active, affirming stance that aligns with the concept of “body functionality,” which has been consistently linked to positive outcomes. The significant residual correlation between ERQ and BAS at W2 suggests a synergistic, reciprocal reinforcement loop that future research should explore causally. This synergy may be explained by a reduction in rumination (a maladaptive emotion regulation strategy often linked to body dissatisfaction) (McComb and Mills, 2021). As dance enhances reappraisal skills, it may disrupt ruminative cycles focused on body flaws. Conversely, as body appreciation increases, cognitive resources previously devoted to body surveillance become available for more constructive cognitive tasks, including emotion regulation.

### 4.4 The dynamic system of bidirectional influences

The cross-lagged panel analyses revealed critical bidirectional relationships, painting a picture of a dynamic, self-sustaining system. Findings such as PWB predicting later DEQ and BAS predicting later DEQ support the broaden-and-build theory of positive emotions (Fredrickson, 2001). The positive emotions and body appreciation fostered by dance may “broaden” an individual’s thought-action repertoire, making them more likely to seek out and engage in novel, challenging, and social activities like continued or deeper dance involvement. This “upward spiral” dynamic suggests that initial engagement in dance can seed its own maintenance and intensification, a feature highly desirable for long-term behavioral interventions. This challenges static, unidirectional mediation models and underscores the value of using longitudinal, cross-lagged designs to capture the reciprocal causality inherent in human psychological development.

### 4.5 Addressing inconsistencies and refining the narrative

This study finding that DEQ scores showed a significant decrease over time requires careful interpretation, as it contrasts with the increase in other variables. This may reflect a measurement or sampling artifact rather than a true reduction in experience. For instance, it could indicate a “response-shift bias” where participants, after 6 and 12 months of reflection and exposure to the study’s focus, recalibrate their internal standards for reporting their dance experience more conservatively. Alternatively, it might reflect natural fluctuations in life circumstances that temporarily reduce formal dance engagement for some participants. Several alternative explanations, besides response shift, may account for the observed decline in DEQ scores. Regression to the mean is plausible, as participants with very high initial dance engagement may have naturally drifted toward the average over time. Seasonality or measurement drift (e.g., changes in academic or work schedules across waves) could also affect self-reported dance engagement. Retention bias is unlikely given our attrition analysis, but unmeasured differences between completers and dropouts cannot be entirely ruled out. Importantly, despite the mean decrease, the strong autoregressive path from T1 DEQ to T3 DEQ (β = 0.54, *p* < 0.001) indicates high rank-order stability, supporting the use of baseline DEQ as a valid predictor. Crucially, the predictive power of the baseline DEQ score remained robust, indicating that one’s predisposing level of dance integration is a key predictor of subsequent psychological growth, even if the absolute level of engagement fluctuates. This highlights the importance of considering both trait-like affinity and state-like participation in future research (Geiser et al., 2017).

### 4.6 Theoretical contributions and practical implications

Theoretically, this study integrates SDT, Embodied Cognition, and emotion regulation theory into a cohesive framework for understanding arts-based physical activities. It demonstrates that the benefits of such activities are not generic but are channeled through specific, measurable psychological processes that correspond to their unique qualitative features. Validating a dual-pathway model, it provides a more granular and accurate map of dance’s mechanism of action than previous unitary models. Practically, these findings offer a blueprint for designing maximally effective dance-based interventions in clinical, educational, and community settings. Interventions should be explicitly crafted to activate both pathways: (1) To boost emotion regulation, emphasize improvisation, emotional expression through movement, and verbal processing of the embodied experience. (2) To cultivate body appreciation, focus on functional cues (“feel the strength in your legs”), non-appearance-based feedback, and styles that celebrate diverse body types and movements. The persistence of a direct effect also encourages practitioners to embrace the holistic, potentially non-specific benefits of artistic engagement, community, and musicality inherent in dance.

### 4.7 Limitation and future research directions

The study’s longitudinal design, use of parallel mediation, and control for key covariates and autoregressive effects represent a significant methodological advance over prior cross-sectional work. The choice of a 6-month lag for mediation was strategic, allowing psychological processes to unfold without excessive attrition. Future research should explore different temporal dynamics using more intensive measurement bursts (e.g., experience sampling) to capture micro-processes (state body appreciation, momentary emotion regulation during/after dance) that aggregate into the macro-level, trait-like changes observed here.

Future studies must address limitations to build upon this foundation. First, experimental causal evidence is needed through RCTs comparing dance to active control groups (e.g., aerobic exercise, other arts). Second, research should deconstruct the “dance experience.” Does the social component drive emotion regulation effects more, while the technical mastery component drives body appreciation? Are improvisational forms more potent than choreographed ones? Third, expanding the nomological network is crucial. Investigating downstream behavioral outcomes (e.g., prosocial behavior, health behaviors) and upstream biological correlates (e.g., cortisol, neural connectivity in body representation areas like the insula) would create a more complete biopsychosocial model. Fourth, studies in specialized populations, such as clinical groups with emotion dysregulation (e.g., borderline personality disorder) or body image disturbances (e.g., eating disorders) are essential to test the therapeutic efficacy and specific mechanisms of dance interventions. A methodological limitation is our use of the traditional CLPM, which conflates between- and within-person variance (Hamaker, 2023). The preferred RI-CLPM could not be applied due to resource constraints. Thus, our findings mainly reflect between-person differences. Future research should employ RI-CLPM, though our three-wave design establishes temporal precedence.

## 5 Conclusion

In conclusion, this longitudinal study provides robust evidence that dance experience is prospectively associated with psychological well-being through the dual pathways of subsequent improvements in emotion regulation and body appreciation, conditional on the measured covariates (age, gender, BMI) and autoregressive controls. These findings establish temporal precedence but do not demonstrate causal mechanisms, which would require experimental manipulation or time-varying confounder control. It positions dance as a unique embodied practice that simultaneously trains cognitive-affective skills and transforms the embodied self-concept, initiating positive reciprocal dynamics that can sustain wellbeing over time. By grounding its findings in Self-Determination Theory and Embodied Cognition, this research moves the field from a generic “dance is good” stance to a sophisticated, mechanistic understanding. It argues for the recognition of dance not merely as a physical art form but as a potent, multi-mechanism intervention for promoting mental health and human flourishing. Future research, employing experimental designs and deeper phenotyping of the dance experience, is now crucial to translate these compelling longitudinal associations into targeted, evidence-based applications across diverse populations and settings.

## Data Availability

The raw data supporting the conclusions of this article will be made available by the authors, without undue reservation.
